# The effectiveness of the Incredible Years™ Parents and Babies Program as a universal prevention intervention for parents of infants in Denmark: study protocol for a pilot randomized controlled trial

**DOI:** 10.1186/s13063-015-0859-y

**Published:** 2015-09-02

**Authors:** Maiken Pontoppidan

**Affiliations:** SFI - The Danish National Centre for Social Research, Department for Children and Family, Herluf Trolles Gade 11, 1052 Copenhagen, Denmark; University of Copenhagen, Department of Public Health, Øster Farimagsgade 5, 1014 Copenhagen K, Denmark

**Keywords:** Parenting, Parenting interventions, Early intervention, Early childhood, Infants, Incredible Years™, Randomized controlled trial, Universal intervention, Prevention

## Abstract

**Background:**

Infancy is an important period in a child’s life, with rapid growth and development. Early experiences shape the developing brain, and adverse experiences can have both an immediate and lifelong impact on health and wellbeing. Parenting interventions offered to parents of newborns can support parents in providing sensitive and responsive care, and reinforce healthy development for their infants. This study aims to evaluate the impact of the Incredible Years™ Parents and Babies Program in a universal setting for parents with infants.

**Methods/Design:**

This is a pragmatic, two-arm, parallel, pilot, randomized controlled trial (RCT) where 128 families with newborn infants up to four-months-old are recruited in two municipalities in Denmark. Families are randomized to the Incredible Years Parents and Babies Program or usual care with a 2:1 allocation ratio. The primary outcome is parenting confidence measured after 20 weeks by the Karitane Parenting Confidence Scale and Parental Stress Scale. Secondary outcomes include measures of parent health, reflective functioning, relationship with the infant, and infant development. Interviewers and data analysts are blind to allocation status.

**Discussion:**

This is the first RCT of the Incredible Years Parents and Babies Program, and one of the first rigorous evaluations of a universally offered preventive intervention for parents with infants. The trial will provide important information on the effectiveness of a relatively brief, universally offered parenting intervention for parents of infants, and will also provide information on infant measures, parent recruitment and participation, and implementation of the program, which could inform future trials.

**Trial registration:**

This trial was registered with Clinicaltrials.gov (identifier: NCT01931917) on 27 August 2013.

**Electronic supplementary material:**

The online version of this article (doi:10.1186/s13063-015-0859-y) contains supplementary material, which is available to authorized users.

## Background

Substantial evidence has documented the importance of a child’s experiences in the first years of life, linking adverse experiences in childhood to later conditions in life, such as depression, health problems, drug abuse, teen pregnancy, and delinquency [1–4]. The relationship is cumulative, as greater numbers of stressful life events in early life results in a greater risk of negative outcomes later in life [2, 5]. The quality of the attachment relationship between the infant and their parents is pivotal in the early years and greatly influences the child’s social, emotional, and cognitive development [6]. A secure attachment to the caregiver predicts a healthy development, whereas an insecure attachment is related to later behavior problems and poor peer relations [7]. It is therefore crucial that appropriate parenting interventions are available to families with infants, especially since interventions in early childhood have been shown not only to be effective [8–13], but also to be more effective than interventions later in life, because it is easier to intervene before problems become entrenched [1, 14].

Parent interventions can be either targeted or universal. In a targeted intervention, families are singled out and offered the intervention because they are thought to be at risk and/or in need of help [15]. Universal interventions on the other hand, are directed at all residents in a specific geographic area and no one is singled out for intervention [15]. Universal interventions have both advantages and disadvantages. The main advantages are: that there is no labelling or stigmatization involved, that the quality of the interventions tend to be high because the middle class is involved, and that at-risk families can be identified and offered more help if needed [15, 16]. The main disadvantages are: that universal programs are expensive, the individual benefits tend to be small, and it can be difficult to find overall effects. Further, it might enlarge social inequality if well-functioning families benefit the most from the interventions [15, 16]. A targeted approach is often applied, but relies on correct identification of families in need of support, which is challenging, as screening instruments never are perfectly accurate, and many families with risk factors do well whereas families with no risk factors might experience difficulties [16]. Child behavior problems tend to be normally distributed across the population, and many families experiencing problems would be missed by a targeted approach based on risk factors [17]. A universal population-level approach is therefore needed to be able to best prevent child developmental problems [17–19].

There are, however, only a few trials of parenting interventions adopting a universal approach [20–27]. Only one of these trials is in a group format and is delivered only postpartum: the Toddlers Without Tears program that was recently evaluated in a randomized controlled trial (RCT) in Australia [25, 28]. Even though the trial was powered to detect a small effect size, only modest improvements in parenting risks were found, but no impact on child behavior at follow-up time points of 18, 24, or 36 months was found. The authors concluded that, ‘A brief universal parenting programme in primary care is insufficient to prevent development of preschool externalising problems’ [25]. Compared to the Toddlers Without Tears program, the Incredible Years™ Parenting and Babies Program (IYPB) offers significantly more sessions (eight compared to three), and starts when the infant is younger (preferably between zero and four months compared to eight months). In this pilot trial, a more intensive intervention offered as a universal approach aimed at a community sample of parents with newborns is evaluated.

An important challenge when performing trials with infants is deciding on primary and secondary outcome measures. Often measures yielding important information on how the parent changes over time, such as measures of parent depression, parenting stress, parenting competence or confidence, or parenting practice are used. Observational measures such as the Bayley Scales of Infant and Toddler Development, the Mullen Scales of Early Learning, and the Strange Situation procedure are also frequently used in infant studies. However, despite it being one of the primary areas targeted by parenting interventions, it is not as common to assess infant development, in particular social-emotional development, using parent-report measures. Infant development is arguably the most difficult construct to measure, as it occurs rapidly, dramatically changes within the first years of life, and it is widely influenced by family and culture values [2, 29–31].

Within recent years a few protocols for infant trials have been published [32–37], but none of these are aimed at universal or low risk populations. Given the relatively low numbers of infant program efficacy or effectiveness trials conducted in this emerging field, it is useful for researchers to learn about some of the possible measures that can be used. In addition, it has been common in the published research in the field to only report on measures where there are significant findings, so researchers designing studies do not know which measures to consider or leave out. In the present trial, a wide array of both parent and infant development measures are used, hopefully aiding future researchers to identify appropriate primary and secondary outcomes for trials on infants.

Parent-infant relationships and parenting practices are central to early-onset social-emotional or problem behaviors, such as aggression or disruptive behaviors [3]. Parenting interventions therefore aim to target these two areas and to support parents in providing sensitive and responsive care to their children. Incredible Years (IY) is a parenting intervention with a focus on strengthening parenting competencies and promoting children’s social, emotional, and academic competence. IY was developed by Carolyn Webster-Stratton more than 30 years ago, and offers a range of programs for parents and teachers of children aged from 0 to 12-years-old. The IY programs are used as both universal and targeted interventions in more than 24 countries worldwide.

IY programs have been evaluated in several RCTs and meta-analysis and were found to be effective on both parent and child outcomes [3, 9, 38–45]. A recent meta-analysis of IY interventions for children between three and nine-years-old shows a mean effect size of d = 0.27 for disruptive behavior across informants [38]. Outcomes that were based on parent reporting showed larger effect sizes for targeted approaches (treatment studies d = 0.50) than universal approaches (indicated sample d = 0.20, selective d = 0.13). There were larger effects for children with severe problems. Another recent meta-analysis based mainly on IY studies [9] of children between three and 12-years-old shows standardized mean differences (SMD) of −0.53 for parent reports and −0.44 for independent reports on child conduct problems. Negative or harsh parenting practices were also reduced (SMD: −0.77) and the intervention was cost effective. The only published study on the effects of IY on children younger than three-years-old is an RCT from Wales of the IY Parent-Toddler program that looked at the effects on parental language [46]. The trial indicated positive effects on two out of five language outcomes. Therefore, although there are very positive results of the IY intervention on both parenting and child outcomes for children three-years-old and older, there is very little knowledge on the effects of IY on children younger than three-years-old.

The IYPB is one of the most recent additions to the IY series. It has been evaluated in Wales with a small sample of mothers living in poverty and has demonstrated positive results [47]. The majority of parents appreciated the group format and stated that they had learned how to encourage the babies’ development, and how to develop effective routines and manage coping strategies. The group leaders also found the program rewarding because they saw positive changes in parenting skills and a growing attachment between infant and parents. Both parental mental health and parenting confidence improved significantly over time; for parenting confidence the effect size was 0.61 (Ewans S, Hutchings J, Davies S, Williams M. Short-term benefits from the Incredible Years Group Based Programme delivered to Parents and their Babies in Powys. Forthcoming). The effects of the IYPB have, however, not yet been evaluated in the more rigorous RCT design, and have not been analyzed in a universal setting. This pilot trial is therefore the first RCT of the IYPB and the first RCT of a universal parenting intervention for parents with infants in Denmark.

The pilot trial has multiple aims:

1. To estimate the effects of the IYPB program offered as a universal intervention in Denmark on parent and infant wellbeing, development, and relationships, and to establish parameters for a future definitive trial.
2. To provide information on usability on a wide array of parent and infant measures.
3. To test recruitment procedures and to determine rates of recruitment and consent.
4. To investigate the implementation of and parents’ acceptance of the IYPB in a universal setting.
5. To provide information on the cost of offering the IYPB as a universal preventive program.

## Methods/Design

**Fig. 1:**
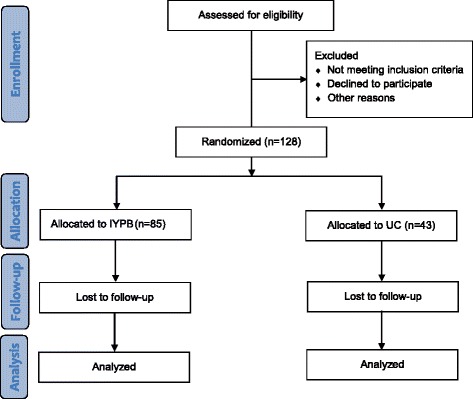
CONSORT trial flow chart. IYPB, Incredible Years Parents and Babies Program; UC, Usual

The trial is a pragmatic, two-arm, parallel, pilot RCT carried out in two municipalities in Denmark. Figure 1 shows the design of the trial. The trial is an external pilot study in the sense that it is a separate trial.

### Eligibility criteria

Eligible participants are mothers with infants living in Ikast-Brande or Herning municipalities in Denmark. Ikast-Brande (population 40,620) and Herning (population 47,765) are bordering municipalities located in central Jutland. If a father is present, he is also invited to participate in the trial.

All mothers in Denmark are entitled to 46 weeks of maternity leave after the birth of a child. The first 14 weeks are exclusively entitled to the mother, but the remaining 32 weeks can be shared with the father. Fathers are entitled to two weeks of parental leave after the birth. This means that almost all mothers participating in the study will be on maternity leave with the child when the intervention is being offered, and will remain on maternity leave until the child is somewhere between six and 12 months. At 12 months, around 81 % of children in Denmark attend daycare [48], so most children will be in daycare at the time of the 18-month follow-up assessment. All municipalities offer free home visits by health visitors to families with newborns and children up to the age of six-years-old. Virtually all families (between 97 and 99 %) take up the offer. Most families receive five to six visits within the first year, but the number is suited to the needs of the family. Families are also offered three child health visits at the general practitioner within the first year after birth [49, 50].

### Inclusion criteria

Mothers with infants aged up to four months who are able to read and write Danish may be included in the trial. In Ikast-Brande, only primiparous (that is, have given birth for the first time) mothers are invited; in Herning primi- and multi-parous mothers living in certain districts of the city are invited.

### Exclusion criteria

Families are excluded if they do not fulfill the inclusion criteria or if they fulfill one or more of the following exclusion criteria: a severe physical or mental disability in parent or child (for example, parents with severe schizophrenia, severe substance abuse, or a child with congenital disease like cerebral palsy) or if the child is placed in out-of-home care.

### Intervention care and comparison

#### Intervention

The families randomized to the intervention group receive the IYPB. The IY programs are based on Bandura’s modeling and self-efficacy theories, Patterson’s social learning model, and Bowlby’s attachment theory. The main foci of the intervention are promoting a warm and nurturing parent–child relationship, enhancing parenting competencies, and encouraging a parent involvement to promote children’s social, emotional, and academic skills and reduce conduct problems [51].

Parents learn through the IYPB how to help their babies feel safe, loved, and secure, and how to promote their babies’ physical, emotional, and language development. The parenting group format stimulates shared learning and peer support networks. Parents practice new skills with babies within the group and are encouraged to try out ideas at home as part of weekly home assignments. Parents also share updates on their infants’ development and activities in a safe and supportive environment [51].

The IYPB uses video vignettes of real-life situations with parents and babies to support the training and to foster discussion in the group. The original American video vignettes are used, with Danish subtitles added. Group leaders also use a Baby Brain Poster with a picture of the brain of a crawling baby to explain to the parents the importance of the development of the infant brain, and how they can strengthen neuron connections and help the brain development of their infant. Parents are furthermore provided with the book *The Incredible Babies* [52], which has been translated into Danish [53]. The book describes how to promote child development and includes a journal section. Before the book was translated, parents were provided with refrigerator notes that included relevant information on, for example, child development or activities the parents could do with their infant to promote child development.

A group is made up of six to eight parents and is led by two trained group leaders. Mothers bring their babies to the sessions, and partners are strongly encouraged to participate. If the mother is single, she can bring a family member such as her mother or sister, if she wants. The program consists of eight sessions of two hours. The parents can arrive half an hour before and eat lunch if they wish. The sessions run every week or every two weeks. The six parts that are covered during the course are: Getting to Know Your Baby; Babies as Intelligent Learners; Providing Physical, Tactile and Visual Stimulation; Parents Learning to Read Babies’ Minds; Gaining Support; and Babies’ Emerging Sense of Self. The group leaders follow a manual to ensure that the intervention is delivered with fidelity [51].

#### Control

The families randomized to the control group receive usual care (UC). UC consists of four to five home visits by health visitors, open consultation hours at a local well-child clinic, voluntary participation in a group of six local mothers, and extra support if needed (for example, extra home visits, family therapy, or video feedback intervention). The intervention group is offered the IYPB on top of UC. UC is consistent with what is offered in the majority of Danish municipalities. The control group families cannot get access to the IYPB, but both they and the intervention group might participate in other infant activities offered by private organizations, such as hymn song at the local church or baby massage classes.

### Procedure

The trial is registered by The National Committee for Health Research Ethics (reference number H-2-2013-FSP60, and has received ethical approval from the Internal Research Council at SFI - the Danish National Center for Social Research. The trial is registered at Clinicaltrials.gov (reference number NCT01931917).

Recruitment is performed by health visitor, social workers, or midwifes in the municipalities. Families are provided with oral information, an information sheet, a two-minute YouTube video (available at www.sfi.dk/godtrivsel) with information on the trial, and a consent form. After receiving the initial consent from the family, the contact person in the municipality sends the contact information to the trial coordinator. An interviewer contacts the mother and sets up an interview in the home. At the T1 visit, written informed consent to participate in the trial is obtained from each mother (and father if he wishes to participate in the trial) and T1 measures are collected. All participants are informed that they can withdraw from the study at any time without their rights being affected.

### Randomization

An independent researcher computes a random allocation list stratified by municipality and with a block size of three. The allocation ratio is 2:1 (IYPB:UC), as it is important for the municipalities to have enough families in the IYPB intervention arm to start the groups. After the baseline assessment is completed, the interviewer informs a designated research administrator that the interview has been completed. The research administrator then randomizes the family by adding the name to the randomization list in the order the names arrive from the interviewer. Then the contact person in the municipality is informed about the allocation status of the family. Participants are informed by their health visitor as to which arm of the study they have been allocated to. In the case where consent to treatment is withdrawn but the participant agrees to remain in the research study, the participant is followed to completion.

### Blinding

Due to the nature of the trial, it is not possible to have a completely blinded design. Participants will know which intervention they are receiving, and the group leaders and health visitors will also know which families are in the intervention arm. Interviewers and coders are blind to group allocation, but participants might reveal allocation status at T2 or T3 assessment. All participants are given an identification number to ensure that the researchers performing the analysis are blinded to allocation status.

### Outcomes

Data are collected at three time points: T1 (baseline), T2 (post-intervention, around four months after baseline), and T3 (follow-up, when the child is 18-months-old). Data collection takes place at the parent’s home at each of the three time points. All interviewers are trained and experienced in carrying out interviews in participants’ homes. The interviewer collects the main part of the background data (for example, education and work status), but the majority of the outcomes are self-reported on computer by the mother. If possible, the father or the partner also completes the questionnaire. Families are compensated by a 200 DKK (approximately 27€) gift card at each visit. The visits are expected to last between 40 minutes (T1) and an hour (T2 and T3). All data are kept at a secure server with password protection. A description of the trial outcomes are outlined below. The timing of the outcomes is shown in Table 1.

**Table 1:** Timing of outcomes

		T1 Baseline	T2 Post-test	T3 Follow-up
Parent measures				
Karitane Parenting Confidence Scale	KPCS	√	√	
Parental Stress Scale	PSS		√	√
Major Depression Inventory	MDI10	√	√	√
World Health Organization Well-Being Index	WHO-5	√	√	√
Rosenberg Self-Esteem Scale	RSS		√	
Being a Mother	BaM-13			√
Parental Reflective Functioning Questionnaire	PRFQ-1			√
Parenting Sense of Competence	PSOC			√
Sense of Coherence	SOC13	√		
Background questions: age, education, occupation, ethnicity, number of children, household status, housing situation, household economy, substance abuse		√	√	√
Single items on parent health, parent life satisfaction, support, and network.		√	√	√
Child measures				
Ages and Stages Questionnaire - Social-Emotional	ASQ-SE	√	√	√
Strengths and Difficulties Questionnaire	SDQ			√
Cognitive Development Questionnaire	CDQ			√
Single items on child health and child temperament		√	√	√
Parent–child measures				
Mother and Baby Interaction Scale	MABISC		√	
Video (15 minutes)	EAS/CARE-Index		√	
Single items on interactions with child			√	√

### Primary outcomes

#### Parenting

##### Karitane parenting confidence scale

The Karitane Parenting Confidence Scale (KPCS) [54, 55] measures parenting confidence for parents of infants aged 0 to 12-months-old. The KPCS consists of 15 items that are rated on a four-point scale (No, hardly ever, No, not very often, Yes, some of the time, Yes, most of the time). A Danish version of KPCS has been developed for the trial by the author and I H Kristensen, and is administered at T1 and T2, but not at T3 since the child is too old at this time.

##### Parenting stress scale

The Parenting Stress Scale (PSS) [56] measures parenting stress and can be used with parents of children up to 18-years-old. The PSS consists of 18 items that are rated on a five-point scale (Strongly disagree, Disagree, Undecided, Agree, Strongly agree). A Danish version of the PSS developed by the author and T Nielsen is administered at T2 and T3, but not at T1 since the items are not considered relevant for parents of newborns.

### Secondary outcomes

#### Parents

##### Major depression inventory

The Major Depression Inventory (MDI10) [57] measures depressive symptoms present within the last 14 days in adults. The MDI10 consists of 10 items that are scored on a six-point Likert scale (All the time, Most of the time, Slightly more than half the time, Slightly less than half the time, Some of the time, At no time). The Danish version of MDI10 is administered at T1, T2, and T3.

##### World Health Organization 5 well-being index

The World Health Organization (WHO)-5 Well-Being Index [58, 59] measures current mental wellbeing in adults. The WHO-5 consists of five items that are scored on a six-point Likert scale (All the time, Most of the time, Slightly more than half the time, Slightly less than half the time, Some of the time, At no time). The Danish version of the WHO-5 is administered at T1, T2, and T3.

##### Rosenberg self-esteem scale

–The Rosenberg Self-esteem Scale (RSS) [60] measures global self-worth in adults. The RSS consists of 10 items that are scored on a four-point Likert scale (Strongly Agree, Agree, Disagree, Strongly Disagree). A Danish version of the RSS is administered at T1 and T2.

##### Being a mother scale

–The Being a Mother Scale (BaM-13) [61] measures a woman’s satisfaction and experience with being a mother. The BaM-13 consists of 13 items that are rated on a four-point scale (No, hardly ever, No, not very often, Yes, some of the time, Yes, most of the time). A Danish version has been developed for the trial by the author. The BaM-13 was created by some of the developers of the KPCS and is developed within the same framework. The BaM-13 is administered at T3.

##### Parental reflective functioning questionnaire

–The Parental Reflective Functioning Questionnaire (PRFQ-1) (Luyten P, Mayes LC, Nijssens L, Fonagy P. The Parental Reflective Functioning Questionnaire: Development and Preliminary Validation. Submitted) measures reflective functioning or mentalization in parents of young infants and children across three domains: pre-mentalizing modes, certainty about mental states, and interest and curiosity in mental states. The PRFQ-1 consists of 39 items that are scored on a seven-point Likert scale (7 Strongly Agree, 4 Neutral or Undecided, 1 Strongly Disagree). For this trial a shorter version with 18 items is used. The 18 items were selected from a Danish version of the PRFQ-1 developed by M S Væver and J Smith-Nielsen. The PRFQ is administered at T3.

##### Parenting sense of competence scale

The Parenting Sense of Competence Scale (PSOC) [62, 63] measures how parents perceive their own competences as a parent. The PSOC consists of 16 items and two subscales: efficacy and satisfaction. The PSOC is scored on a six-point Likert scale (Strongly Agree, Somewhat Agree, Agree, Disagree, Somewhat Disagree, Strongly Disagree). A Danish version of the PSOC developed by A-M Lange and K K Frantzen is administered at T3.

##### Sense of coherence

–The Sense of Coherence (SOC13) [64] measures how people manage stress and stay well within the salutogenic framework phrased by Antonovsky [65]. The SOC13 consists of 13 items that are scored on a five-point Likert scale (Never, Rarely, Occasionally, Often, Always). A Danish version of the SOC13 developed by T Nielsen is administered at T1.

##### Single items

Single items on parent health, parent life satisfaction, support, and network are administered at T1, T2, and T3. Items are scored on an 11-point scale, with 0 representing Worst possible health/Disagree completely/Often and 10 representing Best possible health/Agree completely/Never.

#### Children

##### Ages and stages questionnaire - social-emotional

The Ages and Stages Questionnaire - Social-Emotional (ASQ-SE) [31] measures social-emotional problems and competencies in children aged three months to five years. The ASQ-SE consists of 19 to 33 items that are rated by parents on a three-point scale (Often or always, Sometimes, Rarely or never) and a box parents may check if the behavior is a concern for them. A Danish version based on the experimental version of a second edition of the ASQ-SE has been developed for the trial by the author and is administered at T1, T2, and T3, even though most of the infants will be less than three-months-old at T1.

##### Strengths and difficulties questionnaire

–The Strengths and Difficulties Questionnaire (SDQ) [66–68] measures child behavior and psychopathology in children from 2 to 17-years-old. The SDQ consists of 25 items (five domains: hyperactivity/inattention, peer problems, conduct problems, emotional symptoms, and pro-social behaviors) that are rated by parents on a three-point scale (Not true, Somewhat true, Certainly true). The 2014 revision of the Danish two to four year version is administered at T3. The SDQ is used even though the children are 18-months-old and not 24 months as is the recommended lower age limit.

##### Cognitive development questionnaire

The Cognitive Development Questionnaire (CDQ) [69] measures cognitive development of children from 8 to 24-months-old. The CDQ consists of two sections: section one with 19 scripted games for parents to play with their infant, and section two with 16 items asking about everyday behaviors. Items are rated by parents on a yes/no scale supplemented with information on how many blocks were used. A Danish version of the CDQ has been developed for the trial by the author and is administered at T3.

##### Single items

Single items on child health, temperament, height, and weight are administered at T1, T2, and T3. Child health and temperament are scored on an 11-point scale with 0 representing Worst possible health/Disagree completely and 10 representing Best possible health/Agree completely.

#### Relationship

##### Mother and baby interaction scale

The Mother and Baby Interaction Scale (MABISC) [70, 71] measures the mother-infant relationship. The MABISC consists of 10 items that are scored on a five-point Likert scale (Always, Most of the time, Occasionally, Not often, Never). A Danish version of the MABISC has been developed for the trial by the author. The MABISC is administered at T2.

##### Video

A 15-minute video of the mother and baby is recorded at T2 to assess the mother-infant relationship. The mother is instructed to be with her child on a mat on the floor and to interact with her child as she normally would. The 15-minute video consists of the following phases: six minutes of free play, four minutes of frustration where the child is given a toy that is challenging, 30 seconds of separation, and three minutes of reunion. The videos will be coded within either the Emotional Availability Scales (EAS) system [72] or the Care Index system [73].

##### Single items

Single items measuring parent/child interaction such as singing songs, dancing, and telling stories are administered at T2 and T3. Three single items are administered at T2. One is scored at an 11-point scale with 0 representing Disagree completely and 10 representing Agree completely, and the other two are scored by marking how many days a week the activity happened. At T3, 14 items are administered. These 14 items were adapted from the evaluation of the Preparing for Life program [74], and are scored on a six-point scale (More than once a day, About once a day, A few times a week, A few times a month, Rarely, Not at all).

### Background questions

Background questions and socio-demographics are collected at T1, T2, and T3. They measure parent age, education, occupation, ethnicity, number of children, household status, housing situation, household economy, substance abuse, child birth weight, child gestation at birth, and child health.

### Recruitment and participation

All levels of parent recruitment and participation will be examined. This includes recruitment procedures (information leaflets, YouTube video, challenges and barriers for the health visitors), consent rates, intervention uptake, and mother and father participation in the IYPB sessions. Parent satisfaction with the IYPB is measured by a questionnaire, and a qualitative study looking into how parents experience participation in the IYPB as a universal prevention will be performed.

### Implementation and treatment fidelity

During the trial, a qualitative study of the implementation of the IYPB in one of the municipalities will be performed to look into challenges and successes experienced when moving from using the IYPB as a targeted program to rolling it out as a universal intervention. Treatment fidelity is measured by session checklists completed by group leaders at the end of each session.

### Future outcomes

If further funding is obtained data will be collected at later time points (such as 36 and 48 months) to look for long term effects and dropout rates. In Denmark, researchers have access to very rich register data at a relatively low cost on central long term outcomes, such as school performance, education, income, hospitalization, diagnoses, prescription drug use, marriage status, and childbirths. These are key outcomes, but they are usually not easily collected without the access to register data. Participants will be followed up on in central registers at Statistics Denmark at later time points (for example when the children are 20 and 30-years-old) to look for long term effects of the intervention on both parent and child outcomes (for example school performance, work status, and diagnoses). Register data can be collected for all participants and can be compared to the full population if needed.

### Data analysis

#### Sample size

Lancaster *et al*. [75] recommended that the sample size of a pilot study be a minimum of 30 participants to be able to estimate parameters. As a small effect size is expected with a universal sample, the size of the pilot trial is 128 mothers. With a sample size of 128, a power of 0.8, and a two-sided alpha level of 0.05, it is possible to detect an effect size of 0.50 (Cohen’s d).

#### Planned statistical analysis

Analyses are performed using the software packages R 3.2.1 and STATA 13, or later versions. The data analyst will be blinded to allocation arm. Analysis and presentation of data will be in accordance with the CONSORT guidelines, in particular the extensions to pragmatic trials [76] and nonpharmacologic interventions [77]. Standard descriptive statistics (means, medians, ranges, standard deviations, frequencies, and percentages) will be used to report demographics, and baseline and outcome scores. Data will be examined for missing data and multiple imputation strategies will be used if necessary. Missing data are, however, expected to be low as data are collected through home interviews and the families are compensated.

Primary and secondary outcomes will be analyzed using multiple regression for continuous outcomes, and logistic regression for binary outcomes controlling for baseline scores where possible. A two-tailed test α = 0.05 will be used and parameters will be summarized using 95 % confidence intervals. If assumptions for parametric analysis are not met, non-parametric tests like the Mann–Whitney *U* test will be used. To account for group or therapist effects, standard errors will be clustered around the group for the parents in the intervention arm.

Analysis will follow intention-to-treat (ITT) principles, but completer analysis (such as complier average causal effects (CACE) based on treatment received will also be performed. To examine how non-compliance affects results two levels of participation will be explored: parents that have participated in at least three of the eight sessions, and parents that have participated in at least six of the eight sessions.

As larger effects are expected in parents who present difficulties at the time of recruitment, separate analysis will be carried out for the following three groups: parents who have baseline scores in the clinical range of the measures, parents who are scoring within the lowest 25 % of the distribution at baseline, parents who are scoring within the lowest 50 % of the distribution at baseline.

### Cost

The economic evaluation will be a cost-effectiveness analysis comparing costs related to IYPB with UC. The effect (benefit) will be calculated in natural units (improvements in the primary parenting confidence measures), while costs will be calculated in monetary units (Danish Kroner). Information about both setup costs (training, further education, time for meetings, and so forth), and operating costs related to delivering the group sessions (staff time, parental time, transport, and so forth) will be collected and included in the analysis. Unit costs for health and social care resources will largely be derived from local and national sources and estimated in line with best practice. If possible, average costs of UC in each municipality and across municipalities will be calculated.

## Discussion

This protocol describes a pilot RCT comparing a universal parenting intervention for parents of infants with UC. Infants are dependent on their parents and the quality of their parenting skills, and it is therefore important to support the development of parenting skills in new parents, since lack of parenting skills can have detrimental and long term effects on the infants, such as school failure, behavior problems, relationship problems, substance abuse, and delinquency.

Many parent interventions are expensive because they are intensive and/or long and are offered on an individual one-to-one basis. A relatively brief eight-session group program like the IYPB can therefore be cost effective and possible to roll out to large numbers of families. The intervention is offered at a universal level, making it possible for all parents to participate in an early parenting intervention in a non-stigmatizing way. This trial will provide information on the cost of offering the IYPB in a universal setting, and also important knowledge on the experience of implementing the IYPB in a universal setting.

To the best of our knowledge, this is the first RCT of the IYPB. It is also among the first rigorous evaluations of truly preventive interventions carried out in a real-world universal setting, and will therefore be a valuable addition to the infant intervention literature. Apart from providing information on the effects of the IYPB that can be used to inform a future definitive trial, the pilot trial will provide information on parent recruitment and participation in a trial using health visitors employed by the municipality to recruit mothers, as well as information on experiences with implementing a universal prevention intervention. Furthermore, the trial will yield important information on outcome measures that can be used for the planning and development of future infant trials.

## Trial status

The trial started recruiting in August 2013 and is expected to continue recruiting until the end of summer 2015.

## Additional file

Additional file 1:
**Standard Protocol Items: Recommendations for Interventional Trials (SPIRIT) checklist.** (PDF 57 kb)

## Abbreviations

ASQ-SE: Ages and Stages Questionnaire - Social-Emotional
BaM-13: Being a Mother Scale
CDQ: Cognitive Development Questionnaire
EAS: Emotional Availability Scales
IY: Incredible Years
IYPB: Incredible Years Parents and Babies Program
KPCS: Karitane Parenting Confidence Scale
MABISC: Mother and Baby Interaction Scale
MDI10: Major Depression Inventory
PRFQ-1: Parental Reflective Functioning Questionnaire
PSOC: Parenting Sense of Competence Scale
PSS: Parenting Stress Scale
RCT: Randomized Controlled Trial
RSS: Rosenberg Self-esteem Scale
SDQ: Strengths and Difficulties Questionnaire
SMD: Standardized mean differences
SOC13: Sense of Coherence Questionnaire
UC: Usual Care
WHO-5: World Health Organization Well-Being Index
